# mHealth for Smoking Cessation Programs: A Systematic Review

**DOI:** 10.3390/jpm4030412

**Published:** 2014-07-18

**Authors:** Koel Ghorai, Shahriar Akter, Fatema Khatun, Pradeep Ray

**Affiliations:** 1 Asia-Pacific Ubiquitous Healthcare Research Centre (APuHC), Information Systems, Technology and Management, Australian School of Business (ASB) Quadrangle Building 1039, The University of New South Wales, Sydney NSW-2052, Australia; E-Mails: koel.ghorai@gmail.com (K.G.); f.khatun@student.unsw.edu.au (F.K.); p.ray@unsw.edu.au (P.R.); 2 School of Management, Operations and Marketing, Faculty of Business, University of Wollongong, Wollongong NSW-2522, Australia; 3 School of Public Health and Community Medicine, The University of New South Wales, Sydney NSW-2052, Australia

**Keywords:** mHealth, smoking cessation, service design

## Abstract

mHealth transforms healthcare delivery around the world due to its affordability and right time availability. It has been used for delivery of various smoking cessation programs and interventions over the past decade. With the proliferation of smartphone usage around the world, many smartphone applications are being developed for curbing smoking among smokers. Various interventions like SMS, progress tracking, distractions, peer chats and others are being provided to users through smartphone applications. This paper presents a systematic review that analyses the applications of mobile phones in smoking cessations. The synthesis of the diverse concepts within the literature on smoking cessations using mobile phones provides deeper insights in the emerging mHealth landscape.

## 1. Introduction

**Figure 1:**
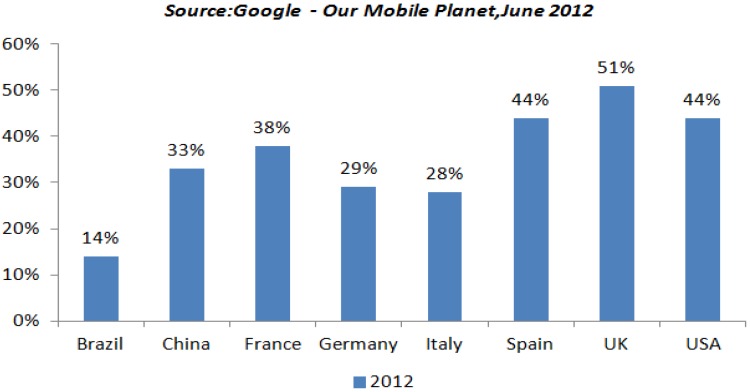
Country wise penetration of Smartphones [13].

Mobile phones have proved to be a ubiquitous mode of communication globally [1]. Currently, mobile phones are owned by almost 80% of the world’s population (see Table 1). More than 6.8 Billion mobile subscriptions are present globally, out of which 1.08 billion are smartphone users [2]. The conversion from generic to smartphones is on a steady rise. Quite a few studies have been carried out on mobile use behavior in the past [3]. Increasing competition among cell phone manufacturers have resulted in a drastic reduction in smartphone prices, which has made it a lot easier for users in low and middle income countries to access them. Smartphones have become one of the most frequently used touch points for Internet access in developed countries. As per the Figure 1, there has been a sharp rise in the penetration of smartphones in countries worldwide with UK leading by 51% as of June 2012. The global mobile phone subscription statistics in Table 1 shows the potential outreach of this platform. In fact, mobile-based messaging systems have already experienced acceptability when backed by the motivation to undertake certain behavior changes. Such interventions range from informational mass-weekly messages to tailor-made customized messages based on user-input.

**Table 1:** Key ICT indicators for developed and developing countries and the world (totals and penetration rates) (ITU 2012).

	(Millions)				Per 100 Inhabitants			
	2010	2011	2012	2013	2010	2011	2012	2013
Mobile-cellular subscriptions								
Developed	1418	1475	1538	1600	115.0	119.0	123.6	128.2
Developing	3901	4487	4872	5235	69.0	78.3	84.3	89.4
World	5320	5962	6411	6835	77.2	85.5	91.2	96.2
Active mobile-broadband subscriptions								
Developed	529	683	788	934	42.9	55.1	63.3	74.8
Developing	249	472	768	1162	4.4	8.2	13.3	19.8
World	778	1155	1556	2096	11.3	16.6	22.1	29.5
Individuals using the Internet								
Developed	830	875	913	958	67.3	70.5	73.4	76.8
Developing	1193	1398	1584	1791	21.2	24.5	27.5	30.7
World	2023	2273	2497	2749	29.5	32.7	35.7	38.8

Mobile phones have been proven effective in delivering interventions for various diseases and health conditions [4,5,6,7]. Mobile messaging services have gained global acceptability for curing diseases [8,9]. Few interventions have been designed to deliver customized motivational messages that lead to smoking cessation through behavior change [4,6,10]. These interventions vary from sending customized motivational messages [10] to multimedia messages [11]. Various online smoking cessation interventions have proved to be effective as well [12]. They provide distraction through games or videos in addition to sending motivational messages through mails. However, some shortcomings were noticed among these interventions. Firstly, the users stopped reading the generic messages after a certain period of time, leading to high participant attrition rates during the intervention. Secondly, accessing web-based services all the time is not possible if it isn’t a Smartphone or a web compatible mobile phone. Thirdly, very few interventions have focused on the intervention design aspect using Smartphones. Fourthly, none of the interventions have included instant (real time) peer support which can have a major effect on quit rates. In addition to this, various government policies have restricted the use of messaging services in many countries and rising cost of telecommunication has made SMS more expensive, thus reducing the chances of mass intervention adoption. Finally, a smartphone based multi-intervention service for smoking cessation is yet to be tested for user acceptance.

Overall, this paper aims to provide a thorough representation of the mobile phone applications in smoking cessation contexts. We organize this article in two main parts. First, we explain the background of smoking prevalence across the world in Section 2. Second, we explain the methodological gestalt and present the results of our systematic review in Section 3.

## 2. Background

### Smoking Prevalence

**Figure 2:**
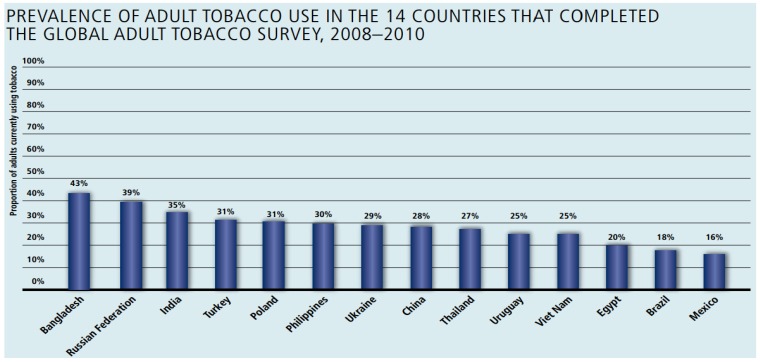
Tobacco prevalence in 14 countries that completed GATS 2008–2010.

More than two thirds of the world’s smokers live in just 10 countries (WHO, 2000)—China, India, Indonesia, Russia, US, Japan, Brazil, Bangladesh, Germany, Turkey (see Figure 2). In China, between 2000 and 2009, the total spending on tobacco quadrupled to US$28.9 billion from US$7.2 billion and in Bangladesh, direct costs of smoking are estimated at US$386 million. Furthermore, between 2003 and 2008, 11.3% of Egypt’s total health expenditure was used to treat tobacco-related illness [14]. Many countries have a very high direct as well as indirect cost to smoking and this is on the rise. At the same time, according to the GATS 2008 to 2010 survey, a large percentage of smokers plan to quit smoking. In developing countries like Bangladesh and India, this is as high as 68% and 47%. In developing countries, such as in India, Smoking causes a large and growing number of premature deaths [15]. According to a study [16], 38.4 per cent smokers—38.3 per cent men and 38.9 per cent women—have made an attempt to quit. In a continent like Australia, of the 4.5 million smokers, 3 million want to quit with about 1 million trying to quit each year. The potential outreach of mobile technology can play a vital role in extending healthcare support and services to populations living in even the remotest of locations.

## 3. Methods, Results and Discussion

The systematic review will help us in analyzing the strengths as well as the limitations of the mobile-based smoking cessation interventions and the gaps present in the literature. In order to establish rigor in the review process, we have followed the guidelines given by Kitchenham [17], which include the following four steps: (a) Resource Identification (b) Selection of studies (c) Data Extraction and Synthesis and (d) Data Analysis.

### 3.1. Resource Identification

As per the experimental method by Dieste [18], we searched for the relevant keywords from Google scholar in the initial step. From the first 370 searches “smoking cessation”, “behavior change”, “RCT”, “mobile interventions”, “Application”, “App” were the key words that were found relevant for the review. These keywords were selected for the successive searches. After finalizing the keywords 15 databases were selected for finding the relevant studies. The databases are Wiley online library, PsycINFO, PubMed, MEDLINE, CINAHL, Web of Science, ERIC, Proquest Science Journals, EMBASE, Informit e-library, Scopus, CochraneDatabase of Systematic reviews, Cochrane Library, Cochrane Central Register for Controlled Trials, Cochrane Methodology Register, Cochrane DSR ACP Journal Club and DARE. The studies published between 1980 and 2013 were considered for the review. Among the keywords selected, search phrases “Smoking Cessation” (And/or) “Mobile” gave the most relevant studies from of the databases. Search was carried out involving multiple combinations of the keywords and 2753 articles came up during the search.

### 3.2. Selection of Studies

Relevant papers were selected from the initial list of searched articles. 2753 articles were found in the initial search. In the first iteration, selection of articles was based on following criteria.

- Would focus on smoking cessation
- Would be peer reviewed
- Would be in English Language only
- Would have mobile phone as one of the modes of communication throughout the intervention

This step gave us 342 articles from which 106 were found to be duplicates. The second iteration had three additional inclusion criteria.

- Study should include at least a randomized controlled trial or quasi-experimental controlled trial
- Should have mobile phone as a primary mode of communication in the intervention
- Behavior change for smoking cessation was one of the major intervention outcomes.

**Figure 3:**
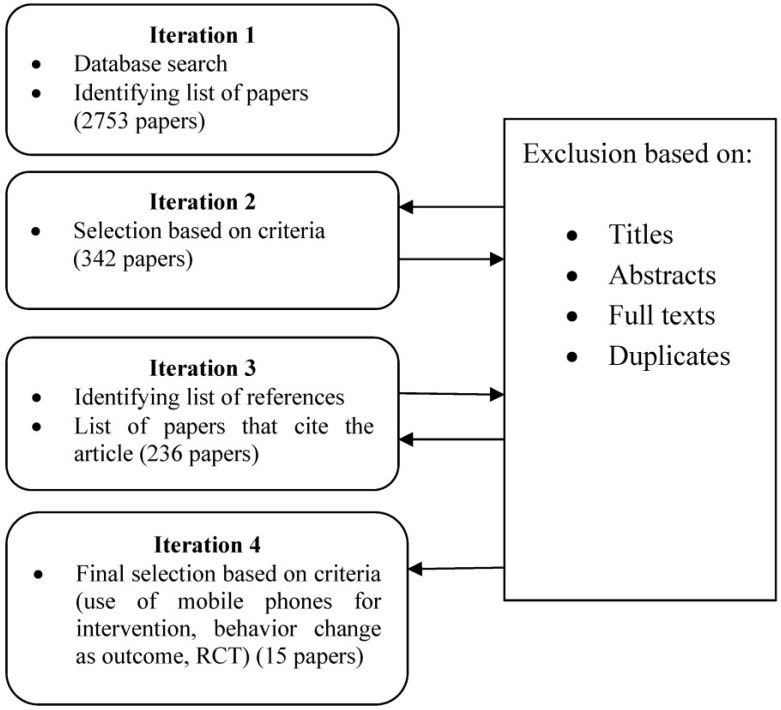
Selection of Studies.

The third iteration included searching on papers that had cited the found papers in iteration one and two. With the additional criteria, and two more iterations, 15 papers were finally selected that adhered strictly to the selection requirements (see Figure 3). The key results have been synthesized in the next paragraph and also listed in Table 2.

**Table 2:** Review of Mobile Based Smoking Cessation Interventions.

Type of Intervention	Description	References
Mode of Smoking Cessation Intervention Delivery	Smoking cessation intervention SMS/Multi Media Messages	[5,10,11,19,20,21,22,23]
	SMS & Internet	[4,6,12,24,25]
	Mobile/Telephone for Telecounselling	[26,27]
Personal Tailoring of SMS	Tailoring of SMS done on the basis of participant name, gender, age, participant history, goals and medical condition	[5,6,10,11,19,20,21,24,25]
User Initiated Craving Support	Quit help lines of motivational messages triggered by a CRAVE or HELP message from the participant	[4,5,19,20,21,25]

### 3.3. Data Extraction and Synthesis

In this step, the papers were thoroughly reviewed and some key information was extracted from each study. The information was grouped into Intervention medium, Location, Participants/sample size, Intervention, Use of tailored messages, Level of interaction, Methodological challenge and any theory if any considered, Duration of cessation outcome measure, Follow up period, Statistically significant increase in cessation, Analysis by racial or minority group and Findings. Of the 15 studies that were reviewed, 11 studies [4,5,11,12,19,20,21,22,23,25,26] were randomized control trials. Four were pre-post design studies. The intervention period ranged from 4 weeks to 1 year. Of the 15 studies, 9 studies used self-reported measures and the rest used bio-chemical validations like determination of salivary cotinine level. Three of the interventions were based on theories which included Social Cognitive Theory, Trans-theoretical model of smoking cessation and Behavioral Self-Regulation Theory.

### 3.4. Characteristics of Studies

Mobile based smoking cessation trials included 13094 participants, with sample sizes ranging from 23 to 5800. Mean age of participants ranged from 16.5 years to 42.8 years. Participation of women in the studies was between 22.1% and 70% except for one study which recruited only women participants. All the studies were conducted in high-income countries like UK, New Zealand, USA (Texas), Norway, Turkey. None of the studies were conducted in developing countries. Most studies had inclusion criteria of participants smoking at least 5 cigarettes a day and some had that of at least 28 cigarettes a day.

### 3.5. Smoking Cessation Interventions

Researchers and IT professionals have developed various kinds of smoking cessation interventions that have firm theoretical grounding. In our review, we will be focusing on mobile-based smoking cessation interventions.

#### 3.5.1. Mobile Interventions

Various smoking cessation programs and interventions have been designed that have been disseminated through mobile phones. Some of the more widely used are

- SMS based quit smoking services
- Tele-counseling
- Multimedia messages based service (Not as widely used and tested as the first two)

From all the articles reviewed, the advantages of mobile phones for the interventions included low cost, better reach, increased interaction between researcher and participants and easier as well as faster way to send tailored and personalized messages [28]. The studies can be divided into three groups based on the type of information sharing and interaction with the participants over the mobile. First, communication through SMS or multimedia messages was seen in eight [5,10,11,19,20,21,22,23] of the studies. Second, messages were communicated through SMS as well as through the Internet in five of the studies [4,6,24,25,27]. Third, use of telephone or mobile for tele-counseling interventions were found in two [26] of the studies. Interventions involving mobile as a medium have the capacity to make communication with ethnic minority groups easier and further the reach of the intervention [20]. This also includes improved engagement and retention of adolescents [6,10,26] through mobile-based smoking cessation interventions. Some of the studies involved personal tailoring of messages [4,5,6,10,11,19,20,21,24]. In some studies tailoring was done on the basis of participant name, gender, age, participant history, goals and medical condition. Few of the studies had user-initiated craving support like quit help lines of motivational messages triggered by a CRAVE or HELP message from the participant [4,5,19,20,21,25]. It was also noticed from the articles that readership of mails was lower and declined substantially as compared to mobile messages whose readership was high and sustained over time [24]. Only one of the studies used performance comparison for motivating the participants for smoking cessation [5]. It used interactive polls and sharing of performance analysis of the participants.

#### 3.5.2. Mobile based Smoking Cessation Intervention Designs

From the systematic survey, it is clear that the interventions have been designed solely on a public healthcare perspective. Most of the interventions have been developed without keeping the end-users or the participants’ feedback or perception in mind. Interventions have been developed and then tested for efficaciousness. Whittaker [29] conducted a study on content development for a multimedia mobile phone based youth smoking cessation intervention. It focused on gathering feedback from participants for content development but not for the service development.

Randomized control trials have been carried out for smoking cessation interventions and that too involving very few participants. Only four of the interventions had a large sample size [11,19,26,27]. It was found that most of the interventions had short term positive behavior change but none of the articles gave any information on long term effects of the interventions. At the same time, researchers should consider a post intervention follow up to determine the long term impact of the interventions. In some of the studies, objective measures were used to assess the intervention outcome. It should be followed more often rather than self-reported measures in future research. It will provide a better validity to the intervention outcomes. Although the SMS based interventions have shown positive behavior change among the participants, still they cannot be deemed as the best intervention mode. A multipronged approach like SMS service in addition to mobile internet applications for group chatting and a system to use patient information for sending messages or chats to support, motivate or distract the patient might give better results. With the development of new technologies and increasing mobile subscriptions worldwide, new ways of message delivery can be designed for reaching out to a larger population. In this context it should be mentioned that during the initial search for the systematic review, we got very few studies that focused on mobile and Smartphone apps for smoking cessation interventions. Although quite a few Smartphone applications have been designed for healthcare management [30] and specially smoking cessation [31], studies are yet to be carried out to measure the impact of these applications. Abroms *et al.* [31] carried out a content analysis of iPhone apps for smoking cessation. Although there are some iphone apps that have been developed to help consumers quit smoking, very few studies have been conducted to measure the outcome of these applications. This can be included in future research for developing mobile/Smartphone apps for smoking cessation.

In the intervention designed by Whittaker [29], video and text messages were developed for participants to view experiences of smoking cessation processes by selected role models. The study tested the reliability of the system and proved the acceptability of the intervention for future studies involving Social Cognitive Theory. At the same time none of the studies talked about self-control theories. According to Baumeister [32], self-control refers to altering one’s own responses and support the pursuit of long term goals. Theory of self-control can be used for designing interventions that tests their impact on behavior change over a long period of time. In the review we also found that most of the studies were carried out in developed countries. There is almost no literature on use of mobile-based smoking cessation interventions in developing countries. The feasibility and acceptability of these interventions in developing countries are yet to be studied and analyzed.

### 3.6. Overall Findings

We focused on medical and social science data base for searching the relevant article. Gray literatures, information on various websites were not included in this review. From the overall review, some gaps were identified. They are listed in the followings:

(1) None of the studies include System Framework/Design component for behavior change services. mHealth applications are integrated with health information systems in advanced countries, where data are shared both by community health workers (CHWs) and clinicians. Although the quality of mHealth services largely depends on the quality of Information Systems (IS)/technology designs, this study found a paucity of research focusing on this stream. Lack of studies on multi-intervention services for behavior change using Smartphones
(2) None of the studies include user acceptance tests of the smoking cessation services.

### 3.7. Discussion

Most of the mHealth services in this review have only focused on SMS based reminder intervention for smoking causation. A multi intervention such as motivational messages through SMS and MMS, social chat, instant peer support, and providing destruction for smoking cessation can be developed and tested [33]. The reason for smoking and personal influences and views to continue and quit smoking may be different from person to person. Health behavior change intervention using socio-cognitive theories have been proven to be effective [34]. For future research, persuasive technologies, especially Persuasive System Designs (PSD) can be recommended for the smoking cessation behavioral change using socio-cognitive theories that involve persuasion and social influence [33,34]. Therefore, a design science framework, social cognitive theories and persuasive technology can be used. It is worth noting that the feasibility and acceptability of the intervention is a pre-requisite for a smart phone based multi-intervention for smoking cessation framework.

### 3.8. Limitations

One of the limitations is small sample sizes. Two of the studies had less than 35 participants. Resluts of these studies may not be generalizable to other populations. Generalization of the results of these studies to other population may not be feasible. All the selected studies focused on mobile-based interventions which has an SMS component. This limited the review to a few studies. Secondly, none of the studies in this review provided cost information. Comparison of this aspect can affect the selection of interventions in the future.

## 4. Conclusions

The review and the taxonomy we propose in this article offer a potentially useful starting point for the development of mHealth applications in smoking cessation programs. With the increase in the number of mobile phone users, researchers can capitalize on mobile technology for designing effective mHealth applications. Future studies can use cross-disciplinary theories for studying behavior change issues in mobile smoking cessation programs.
